# Validation of the German version of the meaning in life measure

**DOI:** 10.1371/journal.pone.0335263

**Published:** 2025-11-17

**Authors:** Albert Anoschin, Johannes Zimmermann, Carina Remmers

**Affiliations:** 1 Department of Psychology, Institute for Mental Health and Behavioral Medicine, HMU Health and Medical University Potsdam, Potsdam, Germany,; 2 Department of Psychology, University of Kassel, Kassel, Germany; De Montfort University, UNITED KINGDOM OF GREAT BRITAIN AND NORTHERN IRELAND

## Abstract

Despite growing interest in meaning in life as a core construct of eudaimonic well-being, there is a lack of brief and validated self-report scales in the German language. We translated the Meaning in Life Measure (MILM) to German and examined its psychometric properties in two studies. The MILM is an 8-item self-report instrument that assesses the experience of meaning in life (MILM-E) and reflectivity about meaning in life (MILM-R) with two subscales. In Study 1 (*N* = 1,189), we confirmed that the German MILM-E and MILM-R load on two positively correlated latent factors, replicating the two-factor structure of the original English measure. In Study 2 we conducted a follow-up assessment (*N* = 300) nine months later, again confirming the fit of the two-factor structure. Additionally, we examined the nomological network of the MILM by relating both subscales to well-being, self-efficacy, satisfaction with life, preference for intuition and deliberation, rumination, and religiosity/spirituality. All hypotheses regarding the direction of associations were pre-registered. As expected, the MILM-E demonstrated strong correlations with concurrent meaning in life measures (*r* > .60) and substantial positive correlations with well-being indicators. The MILM-R correlated positively with search for meaning and rumination but, contrary to our expectations, was not significantly associated with well-being measures. Test-retest correlations over nine months indicated a high temporal rank-order stability of both subscales (MILM-E: *r* = .64; MILM-R: *r* = .59).

## Introduction

In recent years, meaning in life has emerged as a central construct in the study of human well-being [1–3]. The experience of meaning in life is commonly defined as condition of eudaimonic well-being that encompasses having a purpose and striving for self-actualization [4,5]. It is positively related to, but conceptually distinct from hedonic well-being, which is marked by the presence of positive affect and satisfaction with life [6]. Humans possess a fundamental drive to seek meaning in life [7], which encompasses experiences that extend beyond mere feelings of pleasure or cheerfulness [8,9]. For example, people often derive meaning from negative life events as a means of restoring subjective well-being [10,11].

Recent views emphasize that mental health should not merely be defined by the absence of symptoms but rather by the ability to experience meaning in spite of potentially debilitating symptoms [12]. For example, the World Health Organization (WHO) has highlighted subjective well-being as a key determinant of mental health, calling for policies and health promotion programs that enable people to lead meaningful lives [13]. Quantitative research corroborates that experiencing meaning in life is central to well-being because it acts as a protective factor buffering against harmful effects of daily life stressors [14–16]. Similarly, contemporary psychotherapeutic approaches, especially third-wave cognitive-behavioral therapies such as Acceptance and Commitment Therapy (ACT) and Dialectic Behavioral Therapy (DBT) emphasize the role of meaning in life for salutogenesis [17,18]. A shared characteristic of these therapeutic approaches is their orientation toward meaning. They integrate meaning-centered interventions by addressing existential themes, such as the exploration of personal sources of meaning and engagement in value-based action.

Despite this, meaning in life is still rarely assessed systematically in clinical settings (for a notable exception, see [19]). Although several standardized self-report scales for assessing meaning in life are available in English, there is a lack of brief, validated and openly accessible instruments in German, which is particularly striking given the longstanding humanistic and existential traditions of therapeutic approaches like Viktor Frankl’s logotherapy in German-speaking countries. This gap limits our understanding of how meaning in life changes during therapy and what interventions effectively support its growth and maintenance. With the present research, we address this gap by providing a psychometrically sound German adaptation of the Meaning in Life Measure (MILM, [20]). This instrument is intended to support cross-cultural comparisons and empirical research in diverse applied and clinical contexts. In the following sections, we review the current tripartite conceptualization of meaning in life, summarize existing measurement approaches, explain our rationale for selecting the MILM, and outline our validation procedure.

### Tripartite framework of meaning in life

Scholars have long regarded meaning in life as a multifaceted construct [21–23]. However, establishing a consistent definition has been challenging because the answer to what a meaningful life is depends on heterogeneous individual and cultural value systems that ascribe meaning to different phenomena [2,5]. Recently, a tentative consensus has been reached, stating that three distinct but intertwined facets cumulate in the felt sense of meaning in life [3]. This view holds that, in order to experience meaning in life, it is necessary to have a *purpose*, to regard one’s life as *mattering* or being *significant*, and to experience *coherence* with respect to the events in one’s life. Martela and Steger [24] organize these three facets into separate domains, with purpose referring to a motivational drive of pursuing personal goals, mattering/significance referring to the evaluation of one’s life as worth living, and coherence referring to a cognitive “understanding” of one’s life. The latter is established through temporal continuity and the ability to comprehend and connect the events in one’s life into overarching themes or episodes [25,26]. Significance is commonly used synonymously with mattering (e.g., [22]). Martela and Steger [27] discriminate significance, referring to the inherent value of one’s own life to oneself, from mattering of one’s life to the greater scope of the universe. Since their data suggest that both definitions are highly correlated, we regard significance and mattering as two sub-facets of the same dimension and use the terms interchangeably.

### Measuring meaning in life

In the Anglosphere, several self-report instruments have been developed for the assessment of meaning in life, capturing different combinations of theorized facets [22,28–30]. The most widely used measure is the Meaning in Life Questionnaire (MLQ) by Steger et al. [30], which consists of two five-item subscales: The MLQ-Presence (MLQ-P) assessing the presence of meaning, primarily through items querying purpose and coherence, and the MLQ-Search (MLQ-S) assessing the respondent’s active pursuit for meaning in life. Despite its popularity in research, the MLQ has notable limitations. As Hill et al. [20] point out, the MLQ-P subscale does not directly assess the magnitude of subjectively experienced meaning, nor does it cover the mattering/significance facet of the construct. A German translation of the MLQ is publicly available (http://www.michaelfsteger.com/), but it lacks semantic variation. Notably, all items in the German MLQ-P use only one term to refer to the meaning construct (“Sinn”) and potentially obscure semantic nuances that are conveyed in the English version that uses broader vocabulary (e.g., sense of purpose, meaningfulness).

Recently, researchers have sought to refine the operationalization of the tripartite framework by developing self-report scales that aim to empirically separate the three core facets of meaning in life. For example, Costin and Vignoles [28] introduced a 16-item multidimensional meaning scale (MMS) which assesses coherence, purpose, and mattering, along with judgements of overall experienced meaning. Although a four-factor model provided a somewhat better fit than parsimonious one- and three-factor models, the subscales were highly intercorrelated. Subsequent cross-sectional and longitudinal analyses showed limited support for the assumption that coherence, purpose, and mattering serve as distinct predictors of experienced meaning – only mattering consistently predicted overall meaning. Similar issues have been observed with other tripartite instruments, such as the Three Dimensional Meaning Scale (3DM, [27]), where intercorrelations between facet scores often exceed *r* = .70. These findings suggest that measuring the three facets of the tripartite framework separately is challenging and raise the question whether the informational value gained by employing lengthy scales justifies the trade-off in temporal resources and respondent burden. Thus, shorter measures of meaning in life may be sufficient and often preferable, particularly when meaning in life is one of several constructs under investigation.

From this perspective, the MILM developed by Hill et al. [20] offers a compelling balance between parsimony and theoretical grounding in the tripartite meaning framework, making it well-suited for integration into a wide range of research contexts. We therefore considered the MILM an appropriate candidate for translation into German. The measure is comprised of eight items in total, evenly split between an Experience subscale (MILM-E) and a Reflectivity subscale (MILM-R). In previous publications, we used the term “reflection” when referring to the MILM-R. To maintain consistency with the validation study of the original instrument [20], we use the term “reflectivity” throughout this publication. The MILM-E includes one item each for assessing the general felt sense of meaning in life (“I experience my life as meaningful”), significance (“I will be remembered”), purpose (“I have something I want to accomplish in my life”), and coherence (“I can make connections between events in my past and present”). This structure acknowledges the conceptual distinctiveness of the three core facets of meaning but treats them pragmatically as indicators of a single underlying meaning in life factor. In the original validation study, the MILM-E explained additional variance in subjective well-being and depression beyond the MLQ-P, suggesting that the introduction of a significance item and a direct assessment of experienced meaning enhances measurement quality [20].

The MILM-R complements the assessment of experienced meaning by capturing an individual’s tendency to reflect on meaning-related themes (sample item: “There are times in my life when I think about what it all means”). While reflectivity and experienced meaning are distinct constructs, they are theoretically linked. Deliberate reflection is understood as a cognitive pathway to experiencing meaning in life [26,31] and is recognized as one integral process in the meaning-making literature [10]. At the same time, experienced meaning, as captured with the MILM-E, may also emerge from more intuitive, activity-based, or experiential sources [32–34]. Although reflectivity has received limited empirical attention within meaning in life research, it is well established that the way individuals process and reflect on their experiences impacts well-being and mental health [35]. The MILM-R may offer valuable insights into how differences in reflectivity shape the experience of meaning in life [36] and enable researchers to relate these effects to other predictors of meaning in life.

### Overview of studies

We report two studies that were part of a larger research effort investigating associations between intuition, meaning in life, and psychopathology [36,37]. The research project comprised a longitudinal recruitment strategy with three data collection waves (T1, T2, and T3). In Study 1, which served as baseline assessment (T1), we report the translation of the MILM and the evaluation of its factor structure in a sample of *N* = 1,189 participants. Our aim was to replicate the two-factor structure of the original English version by conducting confirmatory factor analyses (CFA) and evaluating model fit. Remmers et al. [37] analyzed other data collected within the participants of Study 1 to address research questions about intuitive performance on a behavioral task.

Study 1 addresses distinct research questions and reports novel analyses of the German MILM at the item level, which have not been published previously. Participants from Study 1 (T1) were invited to a follow-up assessment six months later (T2), which included the MILM and other self-report measures [36]. Data from T2 were used to investigate longitudinal associations between MILM scores and psychopathological symptoms, with results published in Anoschin et al. [36]. Participants who completed T2 (*N* = 542) were then invited to take part in another follow-up (T3) conducted three months after T2.

In Study 2, we report findings from T3 (*N *= 300). Study 2 aimed to replicate the best fitting factor solution derived in Study 1 and to evaluate the construct validity of the two MILM subscales by testing a nomological network of hypothesized associations with related psychological construct. Therefore, the data collection wave T3 for Study 2 included various concurrent measures of meaning in life and other psychological constructs along with the MILM. The hypotheses, data collection procedures and analyses reported in this publication were pre-registered on the OSF (https://osf.io/5b6rp).

## Study 1: Translation and factor structure

### Method

#### Participants.

The MILM data analyzed in Study 1 were collected as part of a larger research effort that investigated associations between intuition, meaning in life, and psychopathology [37]. The research project was approved by the ethics committee of Freie Universität Berlin (proposal number 005/2019) and conducted in accordance with the Helsinki Declaration.

Participants were recruited in an online participant pool (www.prolific.com) that assigns a unique identifier to each participant, facilitating recruitment for follow-up assessments. The authors did not have access to personal information that could identify individual participants. Remmers et al. [37] drew on the same sample to investigate other research questions. The demographic characteristics of the sample are reproduced here for clarity. From *N* = 1204 participants who took part in the data collection, 1189 passed the implemented attention check and were retained for analysis. In this sample, age ranged from 18 to 70 years (*M* = 26.2, *SD* = 8.5), and 62% identified as female, 36% identified as male, and 2% identified as non-binary. 38% of the participants had a university degree or advanced technical certificate, 53% had a high-school degree, 7% had a secondary school degree, 2% had a lower secondary school degree, and one person (0.1%) had no school degree.

#### Materials.

JZ and CR translated the MILM to German, compared and reconciled item wordings. The instrument was then back-translated to English with an AI-based translation service (https://www.deepl.com/). The back-translation was reviewed and approved by the author of the original instrument, Clara E. Hill (see Table A in S1 File). The German MILM is freely available at the Open Science Framework (OSF) repository (https://osf.io/a24zg/). It is comprised of two subscales with four items each. The MILM-E subscale assesses the level of experienced meaning in life. The MILM-R subscale assesses the level of reflectivity about meaning in life. Items are rated on a 9-point Likert scale with verbal markers in the middle (5 = “neutral”) and at the endpoints (1 = “strongly disagree”; 9 = “strongly agree”).

#### Procedure.

Participants performed a behavioral task (judgement of semantic coherence task, [38]) and answered a set of self-report instruments presented in random order. The instruments included the German MILM, the Patient Health Questionnaire-8 for assessing depressive symptoms (PHQ-8, [39]), and the Level of Personality Functioning Brief Form (LPFS-BF 2.0, [40]) for assessing personality functioning impairments. The data collection procedure is described in more detail by Remmers et al. [37]. For the present study, only item-level MILM data were analyzed. We performed CFAs to compare the fit of the two-factor model with correlated factors as established by Hill et al. [20] against an orthogonal two-factor solution and a simple one-factor solution. Analyses were performed using R Studio [41] with the lavaan package [42]. CFA models were computed using maximum likelihood estimation with robust standard errors and Satorra-Bentler correction of test statistics to account for non-normally distributed data. Deviating from the pre-registration, we evaluated model fit by comparing the derived fit indices against the dynamic cutoffs that were computed for each CFA model using the dynamic package for R [43]. The computed cut-offs corresponded to a “level 1” misspecification according to Wolf and McNeish [44], which implies an omission of one cross-loadings (in multi-factor models) or the omission of residual correlations for one-third of the items (in one-factor models). Applying the cutoffs would reject 95% of misspecified models at this level while maximally rejecting 5% of correctly specified models.

### Results

Using CFA, we first compared model fit of three latent factor models. Model 1 specified that items of both MILM subscales load on a single latent factor. Model 2 specified an orthogonal two-factor solution with four MILM-E items loading on the Experience factor and four MILM-R items loading on the Reflectivity factor. Model 3 allowed for correlation between the two latent factors. Results of the CFAs are summarized in Table 1. χ^2^ tests were significant for all tested models, indicating non-perfect model fit. The one factor solution (Model 1) was rejected due to poor fit, with CFI, RMSEA and SRMR values falling outside the computed dynamic fit thresholds. For the orthogonal two-factor model (Model 2), CFI = .926 was below the dynamic cutoff (.930) and RMSEA corresponded to the dynamic cutoff value (.101), indicating overall insufficient model fit. A scaled χ^2^ difference test [45] indicated a better fit of a two-factor solution with correlated factors (Model 3), Δχ^2^(1) = 13.003, p < .001. For Model 3, CFI, RMSEA, and SRMR indices fell within the dynamic thresholds (see Table 1). The correlation between the latent factors Experience and Reflectivity in Model 3 was *r* = .13. Item statistics and factor loadings for this model are presented in Table 2. To estimate reliability, we calculated McDonald’s omega based on CFA results, yielding ω = .75 (95% CI:.73;.78) for Experience and ω = .85 (95% CI:.84;.87) for Reflectivity.

**Table 1:** Robust Chi-square values and fit indices of the CFA models tested in Study 1 (*N* = 1189).

Model	χ^2^	*df*	TLI	CFI	SRMR	RMSEA
One factor (Model 1)	944.236***	20	0.484	0.631 (0.964)	0.171 (0.045)	0.225 (0.058)
Two orthogonal factors (Model 2)	209.702***	20	0.896	0.926 (0.930)	0.088 (0.100)	0.101 (0.101)
Two correlated factors (Model 3)	197.081***	19	0.896	0.930 (0.927)	0.068 (0.095)	0.101 (0.105)
Two correlated factors, without item 6 (Model 4)	95.481***	13	0.933	0.958 (0.910)	0.054 (0.098)	0.080 (0.123)

*Note.* χ^2^ = Satorra-Bentler Scaled Chi-square; *df* = Degrees of Freedom; CFI = Comparative Fit Index; TLI = Tucker-Lewis Index; SRMR = Standardized Root Mean Square Residual; RMSEA = Root Mean Square Error of Approximation. Values in parentheses show computed dynamic cutoffs; adequate model fit is indicated by empirical CFI values above the dynamic cutoffs, and SRMR and RMSEA values below the dynamic cutoffs.

*** p < .001.

**Table 2:** Item statistics and factor loadings of the German MILM in Study 1 (*N* = 1189).

MILM Subscale	Item	Factor Loading	M	SD	Skewness	Kurtosis
Experience	1. Felt Sense	.83	5.61	2.14	−0.31	−0.69
	2. Mattering	.65	5.47	2.09	−0.30	−0.74
	3. Purpose	.62	6.83	2.01	−1.00	0.38
	4. Coherence	.46	6.30	1.77	−0.54	−0.15
Reflectivity	5. Thinking	.73	6.74	1.81	−0.98	0.71
	6. Valuable Topic	.75	6.19	2.10	−0.71	−0.19
	7. Existential Reflection	.72	6.78	1.94	−1.02	0.58
	8. Frequent Reflection	.86	6.30	2.07	−0.75	−0.11

*Note.* Standardized factor loadings are presented for the Experience factor and the Reflectivity factor, respectively, based on Model 3 with two latent correlated factors. The German instrument with complete item wordings can be retrieved from https://osf.io/a24zg/.

Inspection of the modification indices (MI) in Model 3 revealed substantial cross-loadings. Item 6 from the MILM-R subscale (“Meaning in life is a topic I value”) exhibited the largest cross-loading (MI = 92) on the Experience factor, suggesting that its removal would result in the largest improvement of model fit. We tentatively concluded that omitting this item would be theoretically justified because the wordings of the three remaining MILM-R items encompass “thinking” about meaning and thus capture the construct of reflectivity (i.e., deliberate cognitive occupation with meaning in life) more closely than item 6. In a data-driven approach, we computed a fourth model omitting item 6 and allowing correlations between latent factors. This Model 4 yielded the best fit to our data (see Table 1).

### Brief discussion

The evaluation of the German MILM in a large sample demonstrated that it is a reliable self-report measure with acceptable internal consistency for assessing experience of meaning in life (MILM-E) and good internal consistency for assessing reflectivity about meaning in life (MILM-R). A CFA yielded preliminary evidence that the factor structure of the translated instrument is similar to the original English version [20]. The latent factor model with two correlated factors Experience and Reflectivity resulted in good fit to our data. An analysis of modification indices suggested that the differentiation between the two latent constructs could be further improved by dropping item 6 from the MILM-R subscale. However, to avoid overfitting, we opted to retain item 6 in the MILM-R for further studies. In Study 2, we collected follow-up data from *N* = 300 participants of Study 1, aiming to establish construct validity.

## Study 2: Construct validity

In Study 2, our main goals were to replicate the latent factor structure of the German MILM derived in Study 1 and to establish construct validity by examining the convergence and divergence of the MILM-E and MILM-R scales with other psychological constructs. For this purpose, participants completed a battery of self-report questionnaires, including the MILM, two additional meaning in life measures, and instruments assessing cognitive styles and psychological well-being (for a full list, see Table 2). We used a longitudinal recruitment strategy which allowed for an exploratory estimation of the temporal stability of meaning in life reports across three assessment waves, spanning nine months. For this exploratory purpose, we included MILM scores from T1 (Study 1) and T2 [36] into the temporal stability analysis. In the following, the hypotheses are numbered according to their order of appearance in this manuscript; a mapping to the preregistered labelling is provided in Table B in S1 File.

### Confirmation of factor structure

We hypothesized that our revised latent factor Model 4 with omission of item 6 from the MILM-R scale (see Table 1) will yield an adequate fit in the new dataset (Hypothesis H1a), and result in a better fit compared to a one-factor solution (H1b). Although we did not pre-register a hypothesis for the two-factor solution proposed by Hill et al. [20], we also examined the fit of the original two-factor model that retains item 6 (Model 3).

### Establishing convergence with concurrent measures of meaning in life

We aimed to verify the concurrent validity of the German MILM with two established measures of meaning in life, the Meaning in Life Questionnaire (MLQ) by Steger et al. [30] and the multidimensional meaning in life scale (MMS) by Costin and Vignoles [28]. From a substantive perspective, experience of meaning in life as defined by Hill et al. [20] coincides with the “presence of meaning in life” construct proposed by Steger et al. [30]. Hence, we expected a substantial positive association between the MILM-E and the MLQ-P (H2). As a second convergent measure, we chose the coherence, purpose, and mattering items of the MMS [28]. We expected the MILM-E to be positively associated with the MMS (H3) because the MILM-E was specifically designed in accordance with the tripartite meaning framework and includes one item for each facet [20]. We further assumed that reflection about meaning in life (assessed with the MILM-R) is positively related to the search for meaning, captured by the MLQ-S [30]. Whereas the search for meaning refers to a motivational effort directed at finding meaning and focusing on a desirable future state (sample item of the MLQ-S: “I am seeking a purpose or mission for my life”), reflectivity about meaning in life does not necessitate a future-orientation nor a current lack of meaning [31]. However, both scales tap into a deliberate occupation with the topic of meaning in life. Therefore, we expected a significant positive association between the MILM-R and the MLQ-S (H4).

### Testing relationships with measures of hedonic well-being and performative meaning

In accordance with the validation study of the English MILM [20] and a corroborating body of research [46,47], we expected the experience of meaning in life to emerge as a correlate of hedonic well-being. Accordingly, we hypothesized that the MILM-E will be positively correlated with a general index of current psychological well-being (H5) and with satisfaction with life (H6). Going further, we took into account that the presence of meaning in life is not merely a passive state. Meaning may be enacted within work and leisure routines [48], religious or spiritual practice, and through efforts directed at reaching personal goals [24]. Therefore, we assumed that persons who are self-efficacious, that is, confident that they are in control of their behavior and able to attain personal goals [49], would experience more meaning in life compared to those who perceive little self-efficacy (H7). Following the idea that religiosity and spirituality can serve as sources of meaning [50,51], we further hypothesized that the MILM-E will be positively correlated with self-reported religiosity/spirituality (H8).

When formulating our hypotheses concerning reflectivity about meaning in life, we diverged from Hill et al. [20] who found the MILM-R to be positively associated with a compound measure of hedonic well-being, and negatively associated with depressive symptoms. Contrasting these results, we have previously found positive correlations between reflectivity and psychopathology in German speakers [36,37]. A potential explanation for this pattern might be that individuals with a ruminative cognitive style may reflect more on meaning in life. Supporting this idea, C. J. Park and Yoo [52] found that deliberate rumination significantly predicted search for meaning in life, a construct that is theoretically related to reflectivity [31]. Rumination is a well-established predictor of negative outcomes, such as anxiety and depression [35]. Following this train of thought, we hypothesized that the MILM-R would be positively associated with rumination (H9), and negatively associated with psychological well-being (H10) and satisfaction with life (H11).

### Testing relationships with intuition and deliberation

Some scholars have related the meaning in life to intuitive processes, suggesting that the experience of meaningfulness emerges from an automatic detection of coherent patterns, be it in external or internal stimuli [34,53]. For example, humans can detect a common semantic meaning in word triads fast, intuitively and without deliberate conscious processing [54]. Remmers et al. [37] found that self-reported confidence in intuition after performing such tasks was positively associated with the experience of meaning in life. Interestingly, Topolinski and Strack [55] demonstrated that a deliberate approach to solving intuitive meaning detection tasks may even impair performance. Following this line of research, we argue that the experience of meaning in life may arise intuitively and independently of effortful deliberation or explicit knowledge about life’s meaning. We therefore hypothesized that MILM-E will be positively associated with self-reported preference for intuition (H12). Conversely, reflectivity about meaning in life is conceptualized as an act of deliberate cognition. Accordingly, we expected that individuals who tend to reflect more on meaning in life (assessed with the MILM-R) would report a higher preference for deliberation (H13).

### Method

#### Power analysis.

Schönbrodt and Perugini [56] reported that correlation estimates sufficiently stabilize around the true population value with sample sizes of approximately *N* = 250. Accordingly, we considered a target sample size of *N* = 300 as sufficient. A power analysis revealed that, with this sample, a true population correlation of *ρ* = .20 could be detected with a power of.82 using a one-tailed test, with an alpha level of.005 to account for multiple comparisons.

#### Participants.

We invited 542 participants who had previously completed Study 1 and a follow-up assessment (T2, [36]) to participate in the follow-up reported here (T3). Recruitment for T3 ran from 02/06/2022–12/06/2022 via an online panel provider (https://www.prolific.com/) and was automatically terminated upon reaching our target sample size of *N* = 300 participants. The study was performed in compliance with the ethical guidelines for psychological research issued by the German Society for Psychology [57]. Participants were informed in writing about the study’s purpose and procedures. They provided informed consent electronically by ticking a checkbox to confirm their agreement with the terms of participation and the privacy policy. All participants correctly answered the implemented attention check item and were retained for analysis.

The participant’s age ranged from 18 to 68 years (M = 29.7, SD = 9.6), and 61% of participants identified as female, 38% identified as male, and 2 persons (< 1%) identified as non-binary. Education levels among participants were high with 48% reporting a university degree or advanced technical certificate, 43% a high-school degree, 8% a secondary school degree, and less than 1% a lower secondary school degree. We performed a logistic regression analysis to assess whether demographic variables predicted odds of dropout from T1 (Study 1) to T3 (this study). Age emerged as significant predictor of dropout, with older individuals less likely to drop out, *B* = −0.040, *p* < .001. Self-identified gender and education levels were not significantly associated with odds of dropout (all *p*’s > .074).

#### Procedure and measures.

Participants completed the self-report measures listed in Table 3 which were implemented as an online survey. The instruments and the items within each instrument were presented in a randomized order.

**Table 3:** List of instruments used in Study 2.

Construct	Instruments	Authors (English version)	Authors (German version)	Scale description	Sample item ^a^	Expected direction of correlation^b^
Meaning in Life	Meaning in Life Measure (MILM)	Hill et al., 2019 [20]	present publication	MILM-E: Experience subscale, 4 items MILM-R: Reflection subscale, 4 items Agreement rated on a 9-point scale (1 = totally disagree, 9 = totally agree)	MILM-E: “I experience my life as meaningful” MILM-R: “I think about what gives me meaning”	
	Meaning in Life Questionnaire (MLQ)	Steger et al., 2006 [30]	retrieved from www.michaelfsteger.com	MLQ-P: Presence subscale, 5 items MLQ-S: Search subscale, 5 items Agreement rated on a 7-point scale (1 = absolutely untrue, 7 = absolutely true)	MLQ-P: “My life has a clear sense of purpose” MLQ-S: “ I am always looking to find my life’s purpose”	MILM-E ↔ MLQ-P: positive (H2) MILM-R ↔ MLQ-S: positive (H4)
	12 items from the Multidimensional Meaning Scale (MMS)	Costin & Vignoles, 2020 [28]	Translation by CR and AA, reviewed by original author Vlad Costin	Mean score of coherence, purpose, and mattering subscales with 4 items each. Agreement rated on a 7-point scale (1 = strongly disagree, 7 = strongly agree)	Coherence: “I can make sense of the things that happen in my life” Purpose: “I have certain life goals that compel me to keep going” Mattering: “Even considering how big the universe is, I can say that my life matters.”	MILM-E ↔ MMS: positive (H3)
Psychological Well-Being	World Health Organization Well-Being Index (WHO-5)	WHO, 1998 [58]	Brähler et al., 2007 [59]	Five items describing occurrence of appetitive states in the past two weeks rated on a 5-point scale (0 = none of the time, 5 = all of the time)	“Over the past 2 weeks I have felt cheerful and in good spirits”	MILM-E ↔ WHO-5: positive (H5) MILM-R ↔ WHO-5: negative (H10)
Self-Efficacy	Allgemeine Selbstwirksamkeit Kurzskala (ASKU)		Beierlein et al., 2014 [60]	Three items rated on a 5-point scale (1 = does not apply at all, 5 = applies completely)	“I can rely on my own abilities in difficult situations.”	MILM-E ↔ ASKU: positive (H7)
Satisfaction with Life	Satisfaction with Life Scale (SWLS)	Diener et al., 1985 [61]	Janke & Glöckner-Rist, 2014 [62]	Five items rated on a 7-point scale (1 = strongly disagree, 7 = strongly agree)	“The conditions of my life are excellent.”	MILM-E ↔ SWLS: positive (H6) MILM-R ↔ SWLS: negative (H11)
Preference for Intuition and Deliberation	10 items from Preference for Intuition and Deliberation Scale (PID)		Betsch, 2004 [63]	PID-I: Preference for Intuition subscale, 5 items PID-D: Preference for Deliberation subscale, 5 items Agreement rated on 5-point scale (1 = do not agree, 5 = totally agree)	PID-I: “I am a very intuitive person” PID- D: “ Before making decisions I think them through”	MILM-E ↔ PID-I: positive (H12) MILM-R ↔ PID-D: positive (H13)
Rumination	Perseverative Thinking Questionnaire (PTQ)	Ehring et al., 2011 [64]	Ehring et al., 2011 [64]	Occurrence of ruminative thinking queried with 15 items, rated on a 4-point scale (0 = never, 4 = almost always)	“I think about many problems without solving any of them”	MILM-R ↔ PTQ: positive (H9)
Religiosity/Spirituality	One item adapted from the International Social Survey Programme “ISSP 18 – Germany” Religion module		GESIS, 2018 [65]	Level of religiosity/spirituality rated on a 7-point scale (1 = absolutely not religious/spiritual, 7 = deeply religious/spiritual)	“How religious or spiritual would you describe yourself as?”	MILM-E ↔ Religiosity/Spirituality: positive (H8)

*Note.*

^a^In this table, items were translated freely to English for instruments that are only available in German.

^b^Hypotheses are numbered according to their order of appearance in the manuscript. See Table B in S1 File for a mapping to the numbering used in the pre-registration.

### Results

#### Confirmatory factor analyses.

**Fig 1:**
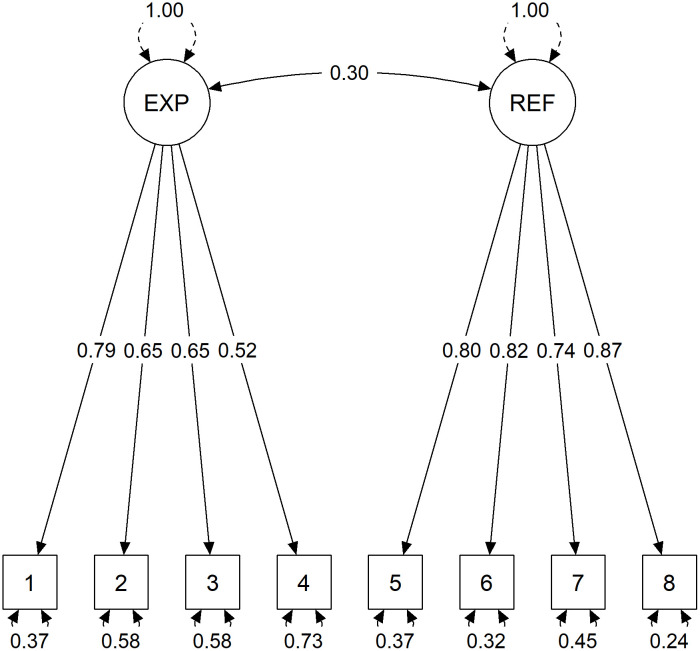
Standardized parameter estimates for latent factor Model 3 in Study 2 (*N* = 300).

Using the data collected in Study 2, we ran the same CFA models as in Study 1. Results of these analyses are listed in Table 4. Coinciding with the results from Study 1, the models with two correlated latent factors (Model 3 and Model 4) showed a good fit to the data, with fit indices ranging within the dynamic thresholds. In contrast, model fit of the one-factor solution (Model 1) was poor. This pattern supported our hypotheses H1a and H1b. In Model 4, which showed the best fit, item 6 from the MILM-R has been omitted. Despite this model’s superior fit, we decided to retain Model 3 as the preliminary best solution in order to avoid further thinning of the construct by removing an item from an already brief measure. A scaled χ^2^ difference test [45] confirmed that Model 3 exhibited a better fit than the one-factor solution (Model 1), Δχ^2^(1) = 102.27, p < .001. Fig 1 illustrates the retained Model 3 with standardized parameter estimates. Model-based reliability estimates were comparable to Study 1 with ω = .76 [95% CI:.71;.80] for Experience and ω = .89 [95% CI:.86;.91] for Reflectivity. Compared to Study 1, the correlation between both factors was larger in magnitude (*r* = .30). Histograms showing the distributions of responses to individual MILM items are provided in Fig A in S1 File.

**Table 4:** Robust Chi-square values and fit indices of the CFA models estimated in Study 2 (*N* = 300).

Model	χ^2^	*df*	TLI	CFI	SRMR	RMSEA
One factor (Model 1)	220.681***	20	0.607	0.720 (0.958)	0.155 (0.056)	0.213 (0.072)
Two orthogonal factors (Model 2)	75.800***	20	0.895	0.925 (0.928)	0.134 (0.108)	0.110 (0.111)
Two correlated factors (Model 3)	61.759***	19	0.915	0.942 (0.940)	0.065 (0.076)	0.099 (0.109)
Two correlated factors, without item 6 (Model 4)	37.802***	13	0.926	0.954 (0.913)	0.064 (0.083)	0.090 (0.138)

*Note.* χ^2^ = Satorra-Bentler Scaled Chi-square; *df* = Degrees of Freedom; CFI = Comparative Fit Index; TLI = Tucker-Lewis Index; SRMR = Standardized Root Mean Square Residual; RMSEA = Root Mean Square Error of Approximation; Values in parentheses show computed dynamic cutoffs; adequate fit is indicated by empirical CFI values above the dynamic cutoffs and SRMR and RMSEA values below the dynamic cutoffs.

*** p < .001.

#### Nomological network of experience and reflectivity.

Table 5 lists zero-order correlations of all measures collected in Study 2. To account for multiple hypothesis testing, we considered *p*-values < .005 based on one-tailed t-tests as significant. Aligning with our hypotheses H2 and H3, we found strong positive correlations of the MILM-E with concurrent measures of meaning in life, namely with the MLQ-P (*r* = .69, *p* < .001) and the MMS (*r* = .74, *p* < .001). Furthermore, we found statistical evidence for all hypothesized associations between the MILM-E and related constructs. Specifically, the MILM-E was positively associated with hedonic well-being, assessed with the general psychological well-being index WHO-5 (H5, *r* = .37, *p* < .001) and the SWLS as a measure of satisfaction with life (H6, *r* = .55, *p* < .001). As hypothesized, the MILM-E was also positively associated with self-efficacy (H7, *r* = .40, p < .001), preference for intuition (H12, *r* = .23, p < .001), and religiosity/spirituality (H8, *r* = .27, *p* < .001).

**Table 5:** Scale statistics, internal consistency, and zero-order correlations of measures in Study 2.

	*M*	*SD*	*α*	1.	2.	3.	4.	5.	6.	7.	8.	9.	10.	11.	12.
1. MILM-E	6.21	1.36	0.74	1											
2. MILM-R	5.92	1.81	0.88	0.28***	1										
3. MLQ-P	4.31	1.35	0.92	0.69***	0.15*	1									
4. MLQ-S	4.37	1.40	0.93	0.05	0.63***	−0.08	1								
5. MMS	4.40	1.14	0.91	0.74***	0.11	0.76***	−0.04	1							
6. WHO-5	49.79^*a*^	20.53	0.89	0.37***	−0.11*	0.51***	−0.23***	0.44***	1						
7. Self-Efficacy	3.82	0.69	0.85	0.40***	−0.04	0.33***	−0.21***	0.38***	0.36***	1					
8. SWLS	4.29	1.28	0.89	0.55***	*−0.03*	0.55***	−0.24***	0.51***	0.53***	0.46***	1				
9. Intuition	3.32	0.70	0.74	0.23***	0.35***	0.16*	0.25***	0.17**	−0.04	0.01	0.10	1			
10. Deliberation	3.71	0.67	0.79	0.10	*0.05*	0.06	0.02	0.08	−0.04	0.23***	0.05	−0.21***	1		
11. Rumination	2.28	0.90	0.95	−0.31***	0.28***	−0.35***	0.32***	−0.38***	−0.44***	−0.36***	−0.36***	0.17**	0.01	1	
12. Religiosity	2.50	1.53	n/a	0.27***	0.21***	0.18**	0.16*	0.29***	0.08	0.04	0.15*	0.17**	−0.10	0.08	1

*Note*. p-values are based on two-sided tests, ^*^p < .05, ^**^p < .005, ^***^p < .001. ^*a*^shows mean WHO-5 percentage score, calculated by multiplying sum scores with 4. Bold correlations are significant at the *p* < .005 level in the hypothesized direction (confirming preregistered hypotheses). Italic correlations are not significant at the *p* < .005 level, contradicting preregistered hypotheses.

Results for the MILM-R subscale were less consistent. In accordance with H4, the MILM-R was positively associated with the MLQ-S (*r* = .63, *p* < .001), supporting the assumed theoretical overlap between reflectivity and search for meaning. Further, data supported the hypothesized association between MILM-R and rumination (H9, *r* = .28, *p* < .001). Contrary to our hypotheses, the MILM-R showed no significant negative associations with indicators of psychological well-being that were assessed with the WHO-5 (H10, *r* = −.11, *p* = .023) and the SWLS (H11, *r* = −.03, *p* = .315). Lastly, we did not find the hypothesized positive association between the MILM-R and preference for deliberation (H13, *r* = .05, *p* = .212).

#### Temporal stability.

Participants who took part in Study 2 (T3) had completed the MILM on two previous occasions, T1 [37] and T2 [36]. Between T1 and T2, the median time difference was 202 days, and between T2 and T3 the median difference was 93.5 days. We analyzed the relative standing of individuals on both MILM scales across time by computing raw and reliability-corrected Pearson correlations across the three assessment waves (see Table 6). In accordance with Bleidorn et al. [66], we interpret these correlations in terms of temporal rank-order stability indices. Descriptively, MILM-E scores (range of *r*’s:.65 to.68) were minimally more stable compared to MILM-R scores (range of *r*’s:.60 to.65). This difference between the scales was more pronounced when considering reliability-corrected estimates (largest *r*_Δ_ = .19) that indicated a comparatively greater rank-order stability of MILM-E scores.

**Table 6:** Rank-order stability of MILM subscales across three assessment waves expressed as Pearson correlations (*N* = 300).

	MILM-E T1	MILM-E T2	MILM-E T3	MILM-R T1	MILM-R T2	MILM-R T3
MILM-E T1	1	0.90***	0.86***	0.19***	0.13*	0.16*
MILM-E T2	0.68***	1	0.90***	0.07	0.24***	0.12*
MILM-E T3	0.65***	0.68***	1	0.14*	0.17***	0.35***
MILM-R T1	0.15***	0.06	0.11	1	0.71***	0.73***
MILM-R T2	0.10	0.19***	0.14*	0.60***	1	0.76***
MILM-R T3	0.12*	0.09	0.28***	0.62***	0.65***	1

*Note.* Values below the diagonal show raw correlations between subscale scores across three assessment waves T1, T2, and T3. Values above the diagonal show reliability-corrected correlations. Reliability correction was performed with the formula $r_{corr}=r\;/\;(\sqrt{\omega_{1}}\cdot \sqrt{\omega_{2}}$ using the CFA model-based reliability estimates from Study 1 where $\omega_{EXP}=0.75\;$and$\;\omega_{REF}=0.85$. Significance tests are based on raw scores, ^*^p < .05, ^***^p < .001.

We also tested for mean-level changes in MILM scores within the sample of *N* = 300 participants who completed all three waves by computing exploratory repeated-measures analyses of variance (ANOVA). No significant mean-level differences in MILM-E scores were found across the three assessment waves, *F*(2, 598) = 2.315, *p* = .100, η² = 0.008, suggesting that the average experience of meaning in life was temporally stable across the examined period. For the MILM-R, the ANOVA indicated a significant effect of time, *F*(2, 598) = 30.424, *p* < .001, η² = 0.092. All Bonferroni-corrected post-hoc comparisons were significant (*p* ≤ .001) and pointed to a reduction in mean levels of reflectivity across the three assessment waves (T1: *M* = 6.59, *SD* = 1.56; T2: *M* = 6.23, *SD* = 1.69; T3: *M* = 5.92, *SD* = 1.81).

## General discussion

In two studies, we examined the latent structure, reliability, and nomological network of meaning in life assessed with the German MILM. CFAs and correlational analyses largely supported the theoretical distinction between Experience (MILM-E) and Reflectivity (MILM-R) as separate dimensions of meaning in life. Our findings confirm that these dimensions can be reliably measured using the German translation of the MILM. As expected, the MILM-E demonstrated strong correlations with concurrent meaning in life measures and indicators of hedonic well-being. The pattern of correlations for the MILM-R did not fully align with our predictions, highlighting the need for further investigations into the interplay between reflectivity about meaning in life and well-being. In the following sections, we discuss the psychometric properties and construct validity of the instrument, outline potential applications and limitations, and interpret the results within the current meaning in life framework, identifying remaining gaps and directions for future research.

### Psychometric properties of the German MILM

The covariance structure between the eight MILM items was best explained by a two-factor solution with correlated factors, supporting the conceptual distinction between an Experience and Reflectivity dimension of meaning in life originally proposed by Hill et al. [20]. Reliability estimates were high for the MILM-R (Study 1: ω = .85, Study 2: ω = .89) but somewhat lower for the MILM-E (Study 1: ω = .75, Study 2: ω = .76). Compared to the English version, the lower reliability of the German MILM-E may have occurred due to a relatively low factor loading of item 2 (Study 1:.46, Study 2:.52), which assesses the sense of coherence regarding past and present experiences. Given the brevity of the scale, its overall reliability can still be considered as sufficient for research at group level and for potential future use in individual assessments [67]. Coherence may be particularly important as a link between reflectivity and the experience of meaning in life [26]. As Hill [31] notes, “[by] making sense of events […], one has a sense of coherence that life makes sense and follows some pattern” (p. 28) and “one needs to reflect […] to have a sense of coherence or to comprehend life” (p. 30). From a theoretical perspective, including all three core facets of meaning in life is essential to capture the construct fully and justifies retaining the coherence item despite its somewhat lower factor loading.

### Construct validity and nomological network

#### Experience of meaning in life.

All pre-registered hypotheses pertaining to the MILM-E as a valid measure of experience of meaning in life were supported. The MILM-E was positively and strongly correlated (*r* > .60) with two convergent measures of meaning in life, the MLQ-P [30], and the MMS [28]. The MILM-E also showed positive correlations with measures of general psychological well-being, satisfaction with life, and self-efficacy. These associations were of lower magnitude (ranging from *r* = 0.37 to *r* = 0.55), suggesting that experience of meaning in life is linked to hedonic well-being, but is not identical to it. Supporting the view that the experience of meaning in life signals high psychological functioning [3], exploratory analyses indicated that the MILM-E was negatively associated with ruminative thinking, an indicator for difficulties in self-regulation.

We further examined associations between the MILM-E and potential sources of meaning in life, namely religiosity/spirituality and intuition. Under the premise that humans possess a natural need for meaning, religiosity and spirituality can provide interpretative frameworks for the coherent organization and understanding of experiences [50]. As predicted, we found a positive association between religiosity/spirituality and the MILM-E. The magnitude of this association (*r* = .27) is comparable to the average effects found in older adults (*r* = .31, Coelho-Júnior et al., 2022). Some scholars propose that the interpretation of experiences as meaningful does not necessitate deliberate processes, but may by large occur intuitively [37,68,69]. Consistent with this view, the MILM-E showed positive associations with self-reported preference for intuition (as hypothesized) and no significant association with preference for deliberation.

#### Reflectivity about meaning in life.

As hypothesized, we found a strong positive correlation between MILM-R and search for meaning (MLQ-S), indicating that reflectivity and an active pursuit of meaning in life are closely related. Consistent with our predictions, reflectivity was also positively associated with rumination. A similar association emerged between search for meaning and rumination, suggesting a potential commonality in the cognitive manifestations of reflectivity and search. However, the MILM-R and MLQ-S exhibited contrasting associations with well-being. In line with previous findings [6], search for meaning was negatively associated with indicators of psychological well-being, including self-efficacy and satisfaction with life. Based on previous findings showing that reflectivity about meaning in life positively predicted depression and personality functioning impairments [36], we expected a similar negative association between reflectivity and hedonic well-being indicators. Contrary to this prediction, we found no significant associations between reflectivity and psychological well-being nor between reflectivity and satisfaction with life. This result contradicts our theorizing that persons who reflect strongly on meaning in life are more prone to ruminative thought and thus, on average, would report lower well-being.

The present findings align with the view of Hill et al. [20] who argued for a conceptual distinction between reflectivity and search for meaning. Notably, reflectivity about meaning in life may be a habit that is upheld even when persons already perceive their lives as meaningful and do not need to engage in further search [31]. This view is corroborated by the positive correlation between the MILM-E and MILM-R in our study, suggesting that reflective processes may enhance the depth of meaningful experiences. However, it is of note that in the present studies, the correlations between reflectivity and measures of experienced meaning were low in magnitude and did not consistently reach the adjusted level of significance. Although it seems uncontroversial that reflectivity is a central pathway to experiencing meaning [10], further research is needed to elucidate the mechanisms through which reflectivity as self-reported in the MILM-R may or may not result in hedonic and eudaimonic well-being.

Interestingly, our hypothesis that individuals who engage in more reflectivity about meaning in life would report a greater preference for deliberation was not supported. Instead, we found a significant positive association between the MILM-R and preference for intuition. This pattern suggests that intuition is not inversely related to reflection about meaning in life. The PID scale [63], which we employed as a measure for intuition, contains items that tap into emotionality and affective decision making (e.g., “Feelings play a big role in my decisions”). It is plausible that persons who prefer to be guided by their emotions and intuitions may also engage in more reflection about the origins or meanings of their actions. It would be interesting to explore the temporal dynamics between affective decisions and reflectivity more closely in daily diary or ecological momentary assessment designs.

### Temporal stability

Analyses of longitudinal data indicated that the average level of experienced meaning in life, as well as the relative standing of individuals on the MILM-E were notably stable over several months. The temporal rank-order stability of MILM-E scores (uncorrected *r *= .65 for a time-lag of approximately 9 months) was higher than the value of *r* = .41 reported for the MLQ-P at a time-lag of 13 months [70]. Rank-order stability estimates for the MILM-E approach commonly reported values for personality traits in adults, although falling slightly below the typically reported range of *r* = .70 to *r* = .75 for test-retest intervals of one year or shorter [66,71,72]. However, this result may be caused by the modest reliability of the MILM-E, as reliability-corrected stability estimates for the MILM-E reach *r* = .90. Overall, our findings suggest that the magnitude of experienced meaning in life may present as a relatively stable trait, with some individuals consistently experiencing more meaning than others.

MILM-R scores showed a similar rank-order stability, but were somewhat less stable than MILM-E scores when using estimates that were corrected for the reliability of the scales. Additionally, we observed significant mean-level changes for the MILM-R across the examined time-period. These findings preliminarily suggest that reflectivity about meaning in life may be more malleable than the experience of meaning in life. Although in absolute terms, differences in experiencing and reflecting on meaning seem to largely persist between individuals across approximately nine months, it is plausible that events in everyday life can influence a person’s global levels of reflectivity and experienced meaning. For example, previous studies showed that transient changes in mood and subtle experimental manipulations of stimulus coherence can significantly alter meaning in life ratings [68,73]. It remains to be determined how stable MILM scores are over even longer periods and how sensitive the measure is to short-term fluctuations. To better capture transient changes in meaning in life and relate them to global assessments, it is advisable to specify the timeframe participants should consider when providing self-reports (e.g., the current moment, the past week, or a general overlook). The use of intensive longitudinal methods, such as ecological momentary assessment (EMA), could then provide insight into how global levels of reflectivity and experienced meaning relate to daily or weekly fluctuations.

### Limitations and future directions

The MILM attempts to provide a standardized and time-efficient method for quantifying the conceptually complex experience of meaning in life with two brief self-report scales. Our findings confirm that the German MILM-E is sufficiently reliable for assessing the experience of meaning in life at group level, although inclusion of additional items could further improve its reliability. In comparison, the MILM-R showed higher reliability estimates, yet the construct of reflectivity requires further conceptual and empirical elaboration. Future research should clarify the optimal level of reflectivity that promotes eudaimonic well-being and explore the potential distinction between adaptive and maladaptive forms of reflectivity. Such work could help to reconcile mixed findings regarding the direction of associations between reflectivity about meaning in life with hedonic well-being and depressive symptoms [20,36]. Since reflectivity has only recently been introduced as a facet of meaning in life, future studies should also investigate how fluctuations in affect, occurrence of life events, and psychotherapeutic interventions influence the extent to which people reflect on meaning in life. To elucidate causal relationships, study designs that experimentally manipulate reflectivity and other variables closely linked to the experience of meaning in life are warranted. For example, it would be interesting to investigate how reminders of meaninglessness affect reflectivity and experience of meaning in life.

The MILM may also prove suitable for individual-level assessments, for example to track changes within individuals in the course of therapeutic interventions [67]. However, the measure’s sensitivity to change must be further established for such applications. In this context, a notable limitation of the present research is our use of convenience sampling, which resulted in a sample whose sociodemographic characteristics deviated from population averages. This limits the generalizability of results to the general population and prevented us from providing normative data. Consequently, the MILM cannot yet serve as a diagnostic tool for identifying critical levels of meaning in life. Establishing norms in the future will be crucial for enabling meaningful individual comparisons and, ultimately, for incorporating meaning in life as an indicator of eudaimonic well-being in healthcare contexts.

The MILM aligns theoretically with the contemporary tripartite framework by covering significance, purpose, and coherence as the three core facets of meaning in life, and extends it with the reflectivity facet. However, we advise using the MILM-E subscale only for global assessments of the experience of meaning in life, as its reliability and validity for assessing individual facets is not established. For researchers seeking more fine-grained assessments of the tripartite structure, our German translation of the MMS [28] may be a promising candidate, though further evaluation of its dimensionality is required. Another option is provided by Martela and Steger [27] with the 3DM, which is not yet available in German, however. A broader question pertains to the practical significance of a nuanced assessments of the three proposed facets. To date, there is limited evidence to support their clear empirical separation [27]. Moreover, several additional facets of meaning in life have been proposed, and it remains to be discussed how these components can be incorporated into a broader theoretical framework. Specifically, it is still unclear whether facets such as belonging [75], experiential appreciation [76], and engagement in meaningful activities [77] should be considered core subcomponents of meaning in life or antecedents of it. It seems plausible that a common g-factor could largely explain the variance in meaning in life judgments across a variety of self-report instruments, but such investigation remains to be undertaken. For now, a parsimonious measurement model that encompasses a general experience factor and an additional reflectivity factor appears sufficient for many applications [20].

## Conclusion

Our translation of the MILM offers a brief and freely available instrument for assessing meaning in life in German-speaking populations. Its psychometric properties are largely consistent with the original English version. The MILM comprises two positively correlated subscales that map onto two distinct latent factors (Experience and Reflectivity). The MILM-E captures the level of experienced meaning in life with four items querying general felt meaning, mattering, purpose, and coherence. Its construct validity is supported by strong correlations with concurrent measures of meaning in life and moderate positive correlations with measures of hedonic well-being and self-efficacy, as well as positive associations with preference for intuition and religiosity/spirituality. The MILM-R comprises four items and reliably assesses the respondent’s tendency to reflect on meaning in life. Reflectivity is positively and strongly associated with search for meaning and moderately associated with experienced meaning (MILM-E). It also shows weaker positive associations with preference for intuition, religiosity/spirituality, and rumination. However, some associations of the MILM-R diverged from pre-registered hypotheses and previously reported results, underscoring the need for further research to situate reflectivity within the broader nomological network of hedonic and eudaimonic well-being.

## Supporting information

S1 File Supplementary tables and figure for the article “Validation of the German version of the meaning in life measure”. (PDF)
