# Supplementary material for: Validation of the German version of the meaning in life measure
Source: PLoS One. 2025 Nov 17;20(11):e0335263. doi: 10.1371/journal.pone.0335263 (PMC12622772; doi:10.1371/journal.pone.0335263)

S1 Supporting Information.

Supplementary Tables and Figure for the Article "Validation of the German Version of the Meaning in Life Measure"

Table A. Back-translation of German MILM Items to English.

Item	German Translation[1]	Back-translation to English[2]
1	Ich erlebe mein Leben als sinnvoll.	I experience my life as meaningful.
2	Ich werde anderen in Erinnerung bleiben.	I will be remembered by others.
3	Ich habe etwas, das ich in meinem Leben erreichen will.	I have something I want to achieve in my life.
4	Ich kann Ereignisse in meiner Vergangenheit und Gegenwart sinnvoll verknüpfen.	I can link events in my past and present in a meaningful way.
5	Ich denke darüber nach, was mir in meinem Leben Sinn gibt.	I think about what gives me meaning in my life.
6	Das Thema "Sinn im Leben" ist mir wichtig.	The topic "meaning in life" is important to me.
7	Es gibt Zeiten in meinem Leben, in denen ich darüber nachdenke, was alles zu bedeuten hat.	There are times in my life when I think about what it all means.
8	Ich denke oft über Fragen nach, die mit dem Sinn im Leben verknüpft sind.	I often think about questions that are related to meaning in life.

Note. [1] The German scale is freely available at https://osf.io/a24zg/

[2] The original English scale is published in Hill, C. E., Kline, K. V., Miller, M., Marks, E., Pinto-Coelho, K., & Zetzer, H. (2019). Development of the Meaning in Life Measure. Counselling Psychology Quarterly, 32(2), Article 2. https://doi.org/10.1080/09515070.2018.1434483

Table B. Mapping of hypothesis labels in the article to labels used in the pre-registration.

Abbreviated hypothesis	Manuscript label	Pre-registration label
Absolute fit of CFA Model 4 (two correlated factors)	H1a	H1a
Better fit of CFA Model 4 (two correlated factors) vs. Model 1 (one factor)	H1b	H1b
MILM-E ↔ MLQ-P	H2	H2a
MILM-E ↔ MMS	H3	H2b
MILM-R ↔ MLQ-S	H4	H2c
MILM-E ↔ WHO-5	H5	H4a
MILM-E ↔ SWLS	H6	H6a
MILM-E ↔ ASKU	H7	H7
MILM-E ↔ Religiosity/Spirituality	H8	H8
MILM-R ↔ PTQ	H9	H5
MILM-R ↔ WHO-5	H10	H4b
MILM-R ↔ SWLS	H11	H6b
MILM-E ↔ PID-I	H12	H3a
MILM-R ↔ PID-D	H13	H3b

Note. The pre-registration is available at https://osf.io/5b6rp
CFA = Confirmatory Factor Analysis; MILM-E = Meaning in Life Measure – Experience; MILM-R = Meaning in Life Measure – Reflectivity; MLQ-P = Meaning in Life Questionnaire – Presence; MLQ-S = Meaning in Life Questionnaire – Search; MMS = Multidimensional Meaning Scale; WHO-5 = World Health Organization Well-Being Index; ASKU = Allgemeine Selbstwirksamkeit Kurzskala; SWLS = Satisfaction with Life Scale; PID-I = Preference for Intuition and Deliberation Scale - Intuition subscale; PID-D = Preference for Intuition and Deliberation Scale - Deliberation subscale; PTQ = Perseverative Thinking Questionnaire; Religiosity/Spirituality = one item adapted from the "ISSP 18 – Germany" Religion module.

Fig A. Response Distributions for MILM Items (Study 2, *N* = 300).

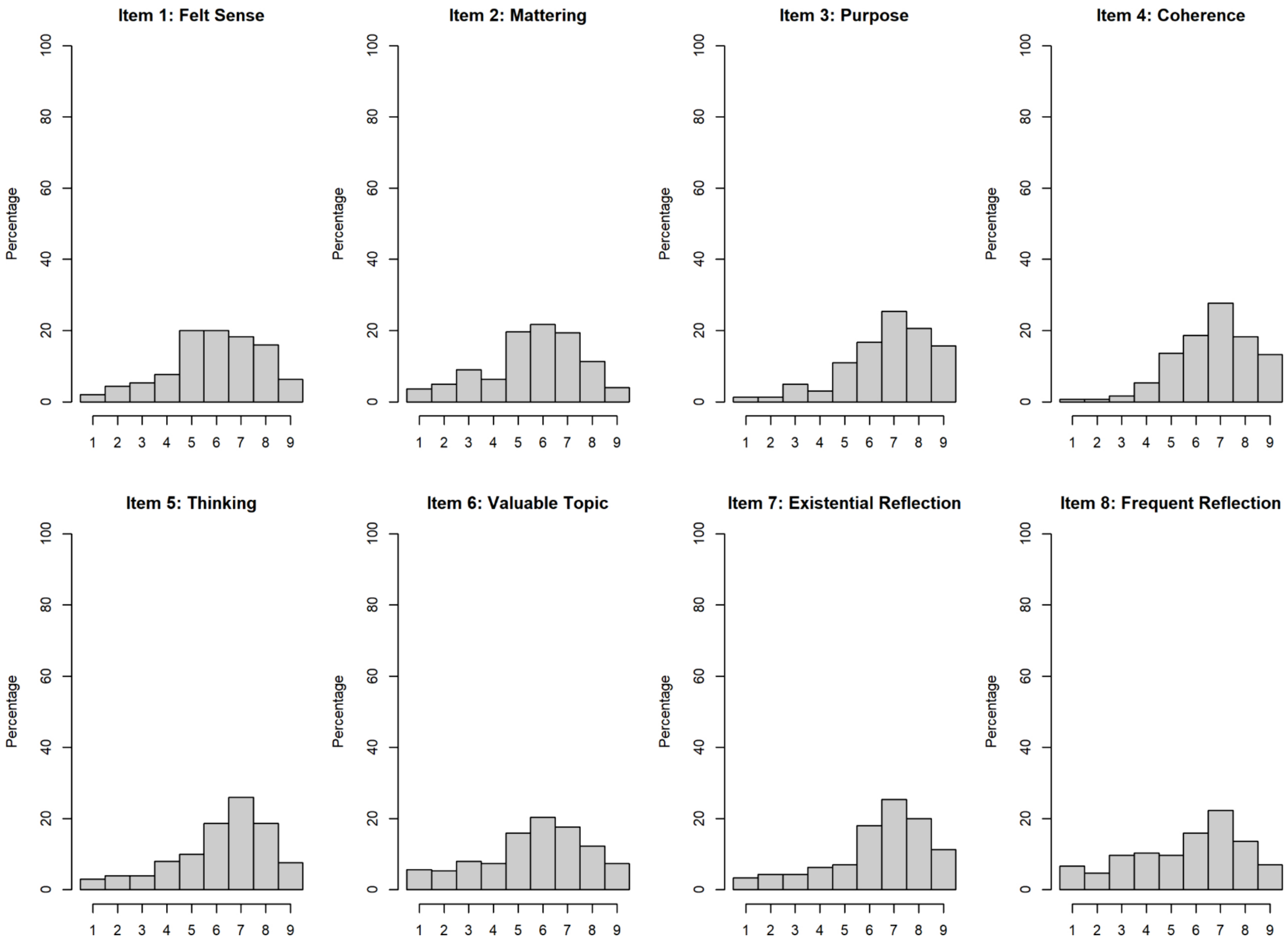

Supplement: S1 File — (PDF) [file pone.0335263.s001.pdf]
